# Novel Recombinase Polymerase Amplification Assay Is Sensitive for Detection of Macrolide Resistance Genes Relevant to Bovine Respiratory Disease Management in Feedlot Calves

**DOI:** 10.3390/vetsci12111079

**Published:** 2025-11-12

**Authors:** Tara Funk, Lianne McLeod, Cheyenne C. Conrad, Rahat Zaheer, Simon J. G. Otto, Cheryl L. Waldner, Tim A. McAllister

**Affiliations:** 1 Western College of Veterinary Medicine, University of Saskatchewan, Saskatoon, SK S7N 5B4, Canada; 2 Lethbridge Research and Development Centre, Agriculture and Agri-Food Canada, Lethbridge, AB T1J 4B1, Canada; 3 HEAT-AMR (Human-Environment-Animal Transdisciplinary AMR) Research Group, School of Public Health, University of Alberta, Edmonton, AB T6G 2G7, Canada

**Keywords:** recombinase polymerase amplification, bovine respiratory disease, macrolide resistance genes, *msrE*, *mphE*, bacterial culture, antimicrobial susceptibility testing, polymerase chain reaction

## Abstract

Macrolides are crucial for the management and treatment of bovine respiratory disease (BRD). However, antimicrobial resistance (AMR) threatens the efficacy of these and other antimicrobials. We developed real-time recombinase polymerase amplification (RPA) assays targeting three clinically relevant macrolide antimicrobial resistance genes (ARGs)—*msrE*-*mphE* and *erm42*—in ≤30 min using extracted DNA. A set of 199 deep nasopharyngeal swabs (DNPS) collected from feedlot calves near the time of arrival were selected based on bacterial culture (BC) results for *Mannheimia haemolytica*, *Pasteurella multocida*, and *Histophilus somni* and antimicrobial susceptibility testing (AST) for tulathromycin, tilmicosin, tildipirosin, or gamithromycin. Samples were also tested for the same targets using RPA and polymerase chain reaction (PCR). In samples that were culture-positive for one or more macrolide-resistant BRD-associated bacteria (n = 101), *msrE-mphE* and/or *erm42* were detected in 95% of cases using RPA. The remaining 98 samples were either culture-negative, or the recovered bacteria were macrolide-susceptible: 43% of these were RPA-positive for at least one macrolide ARG. Together with BC-AST and PCR, Bayesian latent class modelling estimated the clinical sensitivity of RPA for macrolide ARGs to be 95% and specificity to be 58%, with moderate agreement between RPA and BC-AST (κ = 0.52) or PCR (κ = 0.55). The estimated sensitivity of the RPA multiplex assay for the targeted macrolide ARGs was very good, although estimated specificity was limited. However, Sanger sequencing confirmed RPA detection of *msrE-mphE* in BC-AST/PCR-negative samples (n = 23), reflecting the presence of this locus in non-target bacteria, as well as potential ARG variants among BRD bacteria. These findings support the potential of RPA for rapid ARG detection from extracted DNA. Continued assay optimization and evaluation for detection of respiratory bacteria and ARGs will further enhance its diagnostic utility.

## Simple Summary

Bovine respiratory disease (BRD) is a major health concern in feedlot cattle, and macrolide antibiotics are commonly used to treat it. However, the rise in antimicrobial resistance threatens their effectiveness, making it crucial for veterinarians to have fast and accurate tools to guide treatment decisions. Traditional diagnostic methods are often slow and require specialized equipment, limiting their practicality in feedlot settings. This study explored a rapid molecular technique called recombinase polymerase amplification (RPA) to detect three key macrolide resistance genes—*msrE*, *mphE*, and *erm42*—directly from nasal swabs taken from calves shortly after arrival at the feedlot. The performance of RPA was compared to conventional bacterial culture and susceptibility testing and polymerase chain reaction. While RPA showed high sensitivity (95%), its specificity was lower (58%), meaning it detected resistance genes when other methods did not. This was likely due to the presence of these genes in non-BRD bacteria. Overall, RPA offers a promising, rapid alternative for identifying macrolide resistance, but further refinement is needed to improve its accuracy and expand its usefulness to detect a broader range of BRD pathogens and resistance mechanisms in commercial feedlot environments.

## 1. Introduction

Bovine respiratory disease (BRD) is a multifactorial syndrome that is the primary cause of morbidity and mortality in fall-placed, North American feedlot cattle [1,2,3]. An economically important disease, BRD imposes a significant financial burden on producers due to the cost of antimicrobial therapy and metaphylaxis, as well as the losses associated with treatment failure, chronic illness, poor performance, and mortality [4,5].

Respiratory infections associated with BRD are often polymicrobial in nature and involve a complex of bacteria and viruses [6]. Bacterial agents including members of the *Pasteurellaceae* family (*Mannheimia haemolytica*, *Pasteurella multocida*, *Histophilus somni*) and *Mycoplasmopsis bovis* are commensal to the bovine upper respiratory tract, but frequently contribute to BRD. Viral agents, such as bovine respiratory syncytial virus, bovine herpesvirus type 1, bovine parainfluenza-3 virus, bovine coronavirus are also implicated [2,7]. Numerous host and management risk factors, including fall placement of young, lighter-weight calves purchased at auction, contribute to stress and immunosuppression, colonization of the lower respiratory tract, and the development of disease [8,9,10]. Owing to its complex nature, the prevention and treatment of BRD in cattle is challenging [11]. Antimicrobial use remains central to BRD management, where tetracyclines, macrolides, and phenicols are commonly used for disease control and treatment [2,8,11].

As members of the category II (high importance) medically important antimicrobials (MIAs) defined by Health Canada, macrolides are used in both human and veterinary medicine [12]. Tulathromycin (TUL), tilmicosin (TIL), and tildipirosin (TILD) are approved for parenteral use in Canadian cattle to manage and treat clinical BRD [2,8]. In a survey of 36 western Canadian feedlots, macrolides accounted for 41% of the antimicrobials administered for BRD metaphylaxis between 2008–2012 [8]. However, as with any antimicrobial drug (AMD), widespread use of macrolides increases selective pressure for antimicrobial resistance (AMR), threatening their long-term effectiveness [3].

Resistance of BRD-associated *Pasteurellaceae* to macrolides is frequently related to the presence of the antimicrobial resistance genes (ARGs) *msrE*, *mphE*, and *erm42* [2,13,14]. These macrolide ARGs confer resistance via several mechanisms: *erm42* encodes rRNA methylases, which methylate 23S rRNA to mask the antimicrobial binding site of the 50S ribosomal subunit [15,16,17]; *msrE* encodes for a macrolide efflux pump; and *mphE* encodes for a macrolide-inactivating phosphotransferase [16,18].

Horizontal transfer of ARGs on mobile genetic elements (MGEs), such as integrative and conjugative elements (ICEs), further complicates the management of BRD [2]. These transmissible elements can integrate into bacterial genomes and carry ARGs as well as genes involved in virulence, stress response, or heavy metal resistance [19]. Recent studies have shown that BRD-associated *Pasteurellaceae* can harbor ARGs within ICEs [14,20,21] and disseminate ARGs via conjugation [22,23].

Coupled with increasing pressure to minimize antimicrobial use in agriculture, the Canadian Action Plan on Antimicrobial Resistance has recommended that the use of MIAs in livestock production be reduced [24]. To achieve this, beef producers need rapid, accurate diagnostic tools to develop therapies that optimize drug efficacy while minimizing selection for AMR. Recombinase polymerase amplification (RPA) is a rapid, sensitive, DNA-based detection system that requires minimal sample preparation and very little specialized equipment [25]. In contrast to standard diagnostic methods like bacterial culture (BC) and PCR, RPA-mediated DNA amplification occurs typically between 37–39 °C [25,26]. With a time to result of approximately 30 min using extracted DNA, RPA presents an appealing complement to PCR and the industry standard of culture and antimicrobial susceptibility testing (AST) [27]. Since its introduction in 2006, RPA has been employed in various assays to detect infectious agents in human and veterinary medicine [26,27,28,29,30,31].

For RPA tests to be useful for informing antimicrobial use, end-users require estimates of test validity including clinical sensitivity and specificity. Assessing the validity of a diagnostic test has traditionally relied on comparison of the new assay to an existing reference test assumed to be a “gold standard”. Bayesian latent class modelling is a method commonly used to estimate the validity of a diagnostic test when a gold standard test is unavailable [32,33]. Models are used to determine a latent, unknown variable, such as true disease status, which cannot be measured directly. A priori information from the measured outcomes of two or more imperfect diagnostic tests, applied to two or more populations, are used to estimate the sensitivity and specificity of each test, to determine its diagnostic accuracy, or to estimate true disease prevalence.

Herein, a real-time RPA assay was developed and validated for detection of multiple macrolide resistance genes and was used along with previously developed RPA assays to detect BRD-associated bacteria [27] and ICE variants [29] in DNA extracted from nasal swabs obtained from feedlot cattle. The RPA results were compared to quantitative, real-time PCR (qPCR), culture and AST, to estimate the clinical sensitivity and specificity of each test using Bayesian latent class models (BLCMs).

## 2. Materials and Methods

### 2.1. Standardized Bacterial Culture for RPA Assay Development

Strains of *Mannheimia haemolytica* known to contain *msrE*, *mphE*, and/or *erm42* (BioProject Numbers: PRJN1088094, PRJNA181191) based on whole-genome sequencing were used as positive controls in this study (Table 1) [34,35]. Bacterial cultures were grown on Columbia blood agar supplemented with 5% defibrinated sheep blood (Thermo Fisher Scientific, Mississauga, ON, Canada) and incubated at 37 °C for 24 h.

### 2.2. Bacterial DNA Extraction and Preparation of Stock DNA

To generate positive control template DNA, genomic DNA was extracted from *M. haemolytica* cultured cells (~10 µL) resuspended in 200 µL of phosphate-buffered saline (PBS), using the DNeasy Blood and Tissue kit (Qiagen, Toronto, ON, Canada) as per manufacturer instructions.

DNA concentration was measured using a Qubit fluorometer and the Qubit dsDNA BR (Broad-Range) Assay Kit (Thermo Fisher Scientific Inc., Ottawa, ON, Canada). DNA from *M. haemolytica* strains was standardized to 2.0 ng/µL, then diluted to a stock of 20,000–50,000 genome copies per microlitre (gc/µL) and stored at −20 °C for future testing. To estimate copy numbers, the reference genome size of *M. haemolytica* (2.6 Mbp) was used as per Conrad et al. [27].

### 2.3. Conventional RPA Assay Design: msrE, mphE, erm42

Macrolide resistance genes were identified in whole-genome sequences of *M. haemolytica*, *P. multocida*, and *H. somni* using the Pathogen Detection Reference Gene Catalog (National Database of Antibiotic Resistant Organisms, National Center for Biotechnology Information (NCBI), National Library of Medicine (NLM), Bethesda, MD, United States), as well as from our laboratory (Table 1). Three clinically relevant macrolide ARGs (*erm42*, *msrE*, and *mphE*) [14,38,39] were shown to be conserved and were selected as targets for development of RPA assays.

Primer sets for RPA were designed for *erm42*, *msrE*, and *mphE* using the Geneious Prime bioinformatics software platform (version 2020.0.4; Biomatters, Inc., San Diego, CA, USA). Further genomic analysis revealed that *mphE* was consistently located within an operon downstream from *msrE* in the genomes of *M. haemolytica*, *H. somni*, and *P. multocida* [16]. For this operon, primers were designed (Table 2) to span conserved regions of each of the two genes, as well as the intergenic region.

The macrolide ARG targets, *msrE*-*mphE* and *erm42* were each detected in a singleplex assay using conventional RPA, with the TwistAmp^®^ Basic Kit (TwistDx Limited, Maidenhead, UK). A reaction volume of 50 μL was prepared following the conventional reaction protocol [27]. The mastermix was composed of 29.5 μL rehydration buffer (TwistDx Limited, Maidenhead, UK), 11.2 μL nuclease-free water, and 2.4 μL of each forward and reverse primer (final concentration for *msrE*-*mphE* and *erm42* primers: 480 nM) per reaction. The mixture was vortexed and 45.5 μL aliquots were dispensed into 0.2 mL tubes in a strip (eight tubes/strip) containing a lyophilized enzyme pellet (TwistDx Limited, Maidenhead, UK). Genomic DNA of *M. haemolytica* was added at 500 genome copies per reaction (2 µL), followed by 2.5 µL of magnesium acetate (MgAc). Tubes were sealed, vortexed, and briefly spun down using a mini-centrifuge to collect droplets, then placed into the T16-ISO machine (Axxin Ltd., Fairfield, Victoria, Australia) at 39 °C for 30 min. After 4 min, the tubes were removed, manually vortexed, briefly centrifuged again, and returned to the T16 machine for an additional 26 min.

Post-amplification products were purified using a QIAquick PCR Purification Kit (Qiagen, Toronto, ON, Canada) and subjected to electrophoresis on a 2% (*w*/*v*) agarose gel with ethidium bromide at 150 V for 55 min. A fluorescence imager (BioRad, Mississauga, ON, Canada) was used for visualization.

### 2.4. Real-Time RPA Assay Design: msrE-mphE, erm42

Based on the top-performing conventional RPA primer sets, three real-time RPA assays were developed: two singleplex assays for *msrE-mphE* and *erm42*, and one multiplex assay targeting both loci. The singleplex assays were designed to search for potential secondary primer products, which might impede the multiplex reaction, and to determine the expected limit of detection (LOD) and strength of target amplification of each assay without potential competitive inhibition.

A fluorescence probe was designed for each locus (Table 2) using Geneious Prime v2020.0.4 (Biomatters Ltd., Newark, NJ, USA), with probes sized at 48 and 46 nucleotides (fluorescein amidite (FAM) for *msrE-mphE*; dichloro-diphenyl-fluorescein (SIMA) for *erm42*; LGC Biosearch Technologies, Novato, CA, USA). The singleplex reactions used the TwistAmp^®^ exo Kit (TwistDx Limited, Maidenhead, UK), with a reaction mix containing 29.5 µL of rehydration buffer, 11.2 µL nuclease-free water, 2.1 µL of forward and reverse primers (420 nM), and 0.6 µL corresponding probe. The reaction was run on a T16-ISO machine (Axxin Ltd., Eaglemont, VIC, Australia) at 39 °C for 26 min, including four min of initial reaction time, followed by a manual vortex and centrifugation step, then the remaining 22 min of the reaction. A positive identification was defined for either assay as a fluorescence signal threshold of ≥400 mV for at least 60 s. This algorithm was selected to resolve initial false-positive readings in the ‘no template’ control (NTC) of the *msrE-mphE* singleplex assay, which occurred at the original fluorescence threshold (≥200 mV) [27] when “background” fluorescence in the NTC exceeded the threshold, at times to a maximum of ~390 mV. The new algorithm threshold of 400 mV was selected to resolve these false-positive readings with minimal intervention, while also minimizing misclassification of low-positive samples.

To allow for simultaneous detection of both macrolide resistance loci, a multiplex assay was configured using a 45:55 ratio of *msrE-mphE* to *erm42* primers, as initial tests run at a 50:50 ratio showed competitive inhibition of *erm42*. Using the T16-ISO Desktop Application, we also adjusted the fluorescence channel settings to 20% for FAM and 50% for hexachloro-fluorescein (HEX)/SIMA to improve *erm42* detection. Assay validation was completed by running reactions with 29.5 µL rehydration buffer, 10.63 µL nuclease-free water, 210 nM *msrE-mphE* primers, 260 nM *erm42* primers, and aliquots of each probe—73.34 nM (*erm42*) and 60 nM (*msrE*-*mphE*), using the same reaction parameters as for the singleplex assay. The multiplex assay consistently detected both gene targets in the known *erm42*-*msrE*-*mphE*-positive *M. haemolytica* MH44 strain. The known *msrE*-*mphE*-positive, *erm42*-negative MH007 strain [34] was used to ensure assay specificity for *erm42.*

### 2.5. Limit of Detection Testing

Singleplex and multiplex RPA assays underwent LOD testing using serially diluted *M. haemolytica* DNA (10^4^, 10^3^, 500, 400, 200, 10^2^, and 50 genome copies (gc) per reaction). Each dilution was tested up to 30 times (except for the 10^4^ gc/reaction—three iterations, and 500 gc/reaction—six iterations) to determine the LOD in ≥95% of the runs. The LOD for each singleplex and multiplex assay was calculated by Probit regression analysis using SPSS (IBM SPSS Statistics for Windows, Version 26.0, IBM Corp, Armonk, NY, USA), with a step limit of 0.1 and a maximum of 999 iterations. By using a defined and stringent approach to determine the lowest concentration at which *msrE-mphE* and *erm42* could be reliably detected, the LOD of each assay was considered equivalent to the limit of quantitation.

### 2.6. Field Validation of the Multiplex RPA Macrolide ARG Assay

#### 2.6.1. Sample Population

Deep nasopharyngeal swab (DNPS) samples were obtained from feedlot calves in 2020 as previously described [40]. Sampling procedures for this project were approved by an Animal Care Committee (Animal Use Protocol: 20190069; University of Saskatchewan, Saskatoon, SK, Canada) and are described in detail elsewhere [41]. Briefly, high BRD-risk calves were purchased at auction and housed at the Livestock and Forage Centre of Excellence (LFCE) feedlot (University of Saskatchewan, Clavet, SK, Canada) in eight pens of 100 calves per pen. Swab samples were collected from calves at arrival and after 13 and 36 days on feed (DOF). Three nasal swabs were collected per calf, alternating nostrils, and swab heads were cut and placed into a vial containing 3 mL of liquid Amies transport medium (GMP, University of Saskatchewan, Saskatoon, SK, Canada), as previously described [40,42]. Samples were transported to the laboratory at the Western College of Veterinary Medicine (University of Saskatchewan, Saskatoon, SK, Canada) and processed within one hour of sampling [40]. Samples were vortexed for one min, and 300 µL of the raw sample suspension was submitted to Prairie Diagnostic Services Inc. (PDS, Saskatoon, SK, Canada) for bacterial culture and AST. The remaining aliquot was saved at 4 °C for subsequent analyses.

#### 2.6.2. Bacterial Culture and Antimicrobial Susceptibility Testing

Samples were processed as previously described with quality assurance and control procedures [41] for the isolation and identification of *M. haemolytica*, *P. multocida*, and *H. somni*. A 10 µL aliquot of each sample suspension was plated onto Columbia blood agar supplemented with 5% sheep blood (Thermo Fisher Scientific, Mississauga, ON, Canada) to isolate *M. haemolytica* and *P. multocida* and incubated at 35 °C for 42 h. For *H. somni*, samples were cultured on chocolate agar and incubated at 35 °C for 48 h, in a 5% CO_2_ atmosphere. The identity of colonies exhibiting morphology consistent with target pathogens was confirmed using a MALDI-TOF MS Microflex LT instrument (Bruker Corporation, Billerica, Massachusetts, USA) and MALDI Biotyper Microflex LT Compass software (version 1.4).

As was consistent with current surveillance initiatives [43], recent research studies [38], and protocols for diagnostic submissions by the regional laboratory [40], a single, confirmed isolate of each target bacterial species was selected randomly from each sample plate and underwent AST using the Sensititre platform and Bovine BOPO7F AST plate (ThermoFisher Scientific, Waltham, MA, USA). Minimum inhibitory concentration (MIC) breakpoints for *M. haemolytica*, *P. multocida*, and *H. somni* were used according to the Clinical and Laboratory Standards Institute (CLSI) guidelines as previously described in detail with quality assurance and control processes [40].

#### 2.6.3. DNA Extraction

Prior to DNA extraction, bovine DNA was depleted using the HostZEROTM Microbial DNA Kit (Zymo Research, Irvine, CA, USA). Sample suspensions were centrifuged at 6000× *g* for 5 min and the supernatant was discarded, leaving ~100 µL with the pellet. Host depletion solution (500 µL; Zymo Research, Irvine, CA, USA) was added, mixed, and the resulting homogeneous suspension was rotated end-to-end at room temperature for 15 min. Tubes were centrifuged at 10,000× *g* for 5 min and the supernatant was removed. Microbial selection buffer (100 µL; Zymo Research, Irvine, CA, USA) and microbial selection enzyme (0.5 µL; Zymo Research, Irvine, CA, USA) were added, and the pellet was resuspended by vortexing for 5–10 s. Tubes were centrifuged (≤1500× *g*) and incubated for 30 min at 37 °C. Following incubation, 10 µL of proteinase K was added to each sample. The tubes were vortexed for 10 s, centrifuged (1500× *g*), and then incubated at 55 °C for 10 min. Finally, DNA/RNA Shield^TM^ (200 µL; Zymo Research, Irvine, CA, USA) was added to each tube, which was vortexed for 10 s and incubated at room temperature for 5 min to complete depletion of host DNA prior to extraction. Bacterial DNA was then extracted using the DNeasy Blood and Tissue kit (Qiagen, Toronto, ON, Canada) and eluted into 100 µL of elution buffer (Buffer AE, Qiagen, Toronto, ON, Canada).

#### 2.6.4. Inclusion Criteria for Field Samples: Macrolide Resistance

DNA was extracted from 199 DNPS collected from feedlot calves in the fall of 2020 and selected for RPA screening of *msrE*, *mphE*, *erm42*, BRD-associated bacteria, and ICEs (Table 2). The DNA from the 199 DNPS was strategically selected from all available samples to optimize the power of subsequent analyses by targeting a mix of samples with and without evidence of macrolide-resistant bacteria. Inclusion criteria were based on bacterial culture results and phenotypic resistance to TUL, TIL, TILD, and/or gamithromycin (GAM). Samples were divided into two groups: (i) samples with detected “phenotypic-macrolide-resistance-positive” (MacRes+) bacteria (n = 101), where any of *M. haemolytica*, *P. multocida*, and/or *H. somni* were isolated and exhibited phenotypic macrolide resistance, and (ii) samples where no “phenotypic-macrolide-resistance-negative” (MacRes-) bacteria were detected (n = 98). The resistance-negative group included 68 samples from which one or more BRD-associated bacteria were isolated, but were susceptible to macrolides (TUL, TIL, TILD, GAM), and 30 samples from which no BRD pathogens were cultured. Among the BRD-associated bacteria isolated from the MacRes+ samples, the most common resistance pattern was to GAM and TUL (GAM-TUL) in *M. haemolytica* isolates (Table 3).

Resistance to other drugs among MacRes- samples included a single *M. haemolytica* isolate resistant to ampicillin (AMP), and nine *P. multocida* isolates that were resistant to spectinomycin (SPECT) and tetracycline (TET) (Table S1). The six *H. somni* isolates recovered from the macrolide-susceptible samples were pansusceptible.

### 2.7. Experimental Testing

**Figure 1:**
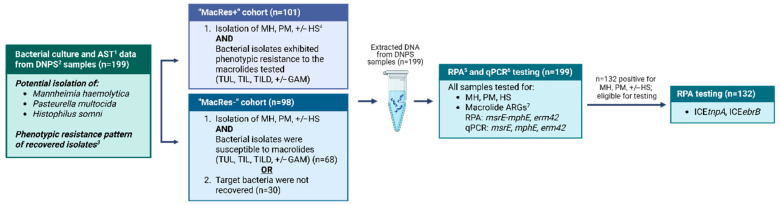
Flowchart of the diagnostic testing strategy used for bacterial culture, antimicrobial susceptibility testing, recombinase polymerase amplification, and quantitative polymerase chain reaction testing of deep nasopharyngeal swabs collected from calves for the detection of bacteria commonly associated with bovine respiratory disease and antimicrobial resistance targets of interest (n = 199). ^1^ AST: antimicrobial susceptibility testing; ^2^ DNPS: deep nasopharyngeal swab. ^3^ Possible phenotypic resistance to antimicrobial drugs included ampicillin, gamithromycin (GAM), spectinomycin, tetracycline, tilmicosin (TIL), tildipirosin (TILD), and tulathromycin (TUL). ^4^ MH: *Mannheimia haemolytica*, PM: *Pasteurella multocida*, HS: *Histophilus somni*. ^5^ RPA: recombinase polymerase amplification; ^6^ qPCR: quantitative polymerase chain reaction; ^7^ ARGs: antimicrobial resistance genes, in this case referring to macrolide ARGs *msrE*, *mphE*, and *erm42.* Only samples that tested positive for *M. haemolytica*, *P. multocida*, or *H. somni* using RPA were tested for mobile genetic element variants ICE*tnpA* and ICE*ebrB*; *Created in BioRender. L, S. (2025) https://www.biorender.com/* accessed on 10 November 2025.

Field samples selected using BC-AST (Section 2.6.2) data, were then tested using RPA and qPCR (Figure 1).

#### 2.7.1. RPA Testing

Reaction setup for RPA testing of DNA from the 199 field samples was analogous to that of the multiplex assay development protocol, with the exception that the volume of extracted template DNA was increased to 10 µL per reaction and nuclease-free water was reduced to 2.63 µL. Additional RPA targets followed the testing protocols established by Conrad et al. [27,29] for bacterial BRD pathogens (*P. multocida*, *H. somni*, and *M. haemolytica* serotypes 1 and 6) [27,44] and ICE complex variants (ICE*tnpA*: *tetH_tnpA*, ICE*ebrB*: *tetH*_*ebrB*; Table 2). Samples were further tested for ICE variants only if at least one BRD bacterium of interest was identified by RPA.

#### 2.7.2. Quantitative, Real-Time PCR Testing

Following RPA, the remaining volume of each of the same 199 extracted DNA samples was tested for the presence of macrolide resistance genes (*msrE*, *mphE*, *erm42*) and key bacterial pathogens (*M. haemolytica*, *P. multocida*, *H. somni*) by the Nebraska Veterinary Diagnostic Centre (NVDC; Lincoln, NE, USA), using multiplex, qPCR assays on the Rotor-Gene Q (RGQ) platform (Qiagen, Hilden, Germany) and protocols outlined by Loy et al. [45] and Dutta et al. [46].

For the detection of macrolide ARGs, DNA extracted from the 199 DNPS samples was tested at NVDC using the RGQ platform and a validated, four-plex qPCR assay targeting *msrE*, *mphE*, and *erm42* individually, as well as *tetR* [46]. For each 25 µL reaction, the PCR mastermix comprised 12.5 µL of 2× QuantiFast multiplex PCR master mix (Qiagen, Hilden, Germany), including 1 µL of each, respective primer-probe mix (forward primer [10 µM], reverse primer [10 µM], probe [10 µM] for the four targets, 4 µL total), 6.5 µL of nuclease-free water, and 2 µL of genomic template DNA. The thermocycling conditions for this assay consisted of 5 min at 95 °C, followed by 45 cycles at 95 °C for 15 s, and 59 °C for 40 s for both the primer annealing and extension steps [46].

A mean Ct threshold of 31.8 for detecting *msrE*, *mphE*, and *erm42* was reported for the qPCR assay using receiver operating characteristic (ROC) curve analysis. The reported sensitivity and specificity of the qPCR assay were 43% and 83%, respectively [46].

Additionally, samples were tested for *M. haemolytica* (all serotypes), *P. multocida*, and *H. somni* using the RGQ platform. For each 25 µL reaction, PCR mastermix was prepared with 10 µL of 2× QuantiFast multiplex PCR master mix (Qiagen, Hilden, Germany), 1.0 µL of primer-probe mix for each target (10 µM, 5.0 µL total), and 5.0 µL of extracted template DNA. The thermocycling conditions included an initial denaturation at 95 °C for 5 min, followed by 40 cycles of 95 °C for 15 s, and 60 °C for 40 s for both the primer annealing and extension steps [45]. A Ct value > 33.4 (*M. haemolytica*, *P. multocida*), or >39.8 (*H. somni*) was designated as negative, or “not detected”, while a value below this threshold was considered positive for the detection of each target bacterium [45] (Supplementary Material).

#### 2.7.3. Sanger Sequencing and Selected Long-Read Metagenomic Next-Generation Sequencing

A subset of 23 samples tested for macrolide ARGs were submitted to the National Research Council (NRC) of Canada (Saskatoon, SK, Canada) for Sanger sequencing (Table 4). The samples were of specific interest because they were presumptive RPA false positives as they were macrolide-resistance-negative by BC-AST, negative for the *msrE* and *mphE* by PCR, and positive for *msrE-mphE* using RPA. Another subset of samples (n = 8) that were RPA positive *erm42*, were tested as these results also differed from BC-AST, and qPCR testing (Table 4).

For sequencing, the designated RPA primers for each target were prepared at 5 pmole/µL (forward and reverse), and the RPA post-amplification samples were normalized to 10 ng/µL. Samples were considered Sanger-sequencing-positive for macrolide ARGs if the primers and/or probe annealed at the correct genetic location in silico using Geneious Prime software (version 2020.0.4), and negative if no primer or probe sequence information could be deduced.

The same subset of 23 suspected RPA false-positive samples submitted for Sanger sequencing for *msrE* and *mphE* were also tested using a previously described protocol for long-read metagenomic next-generation sequencing (mNGS), where ARGs associated with identified bacterial were reported [47]. The objective of this work was to potentially identify the bacterial source of ARGs.

A BLAST search for the amplified RPA *msrE-mphE* target sequence was also completed using the Discontiguous Megablast program (Geneious Prime v2020.0.4) and core nucleotide database (core_nt) framework (NCBI) to align the amplicon with curated bacterial genomes (Table 2). A high, 2000-hit maximum was selected to search for these ARGs in other, non-BRD-related bacteria common to the bovine nasopharyngeal microbiome, which could account for *msrE-mphE* in samples where resistance had not been detected in *Pasteurellaceae*.

Conversely, any database hits resulting in detection of genetic sequences closely resembling, but different than *msrE-mphE* could account for RPA false positives or variants.

### 2.8. Statistical Analysis

Bayesian latent class models were used in the absence of a gold standard reference test to assess clinical test performance of the RPA assays relative to bacterial culture and phenotypic AST, and qPCR for detecting macrolide resistance and BRD-associated bacteria. Three-test and three population models were developed for both macrolide resistance (BC-AST, RPA, qPCR) and specific BRD bacteria (BC, RPA, qPCR). Samples collected at each time point were expected to have varied prevalence and therefore comprised the three populations in the BLCM: 1DOF (n = 97), 13DOF (n = 77), and 36DOF (n = 25). Assumed conditional dependence between RPA and qPCR tests was modeled as covariance. Models were developed and run using JAGS software v4.3.0 [48] and the ‘runjags’ package [49] in R v4.1.0 (R Foundation for Statistical Computing, Vienna, Austria) [50].

For BRD-bacterial-pathogen models, uninformative priors (beta (1, 1)) were used for the sensitivity of each test, the specificity of RPA, and the prevalence in each population. A beta (100, 1.5) distribution was used to model the prior for the clinical specificity of bacterial culture for the detection of each bacterial target based on subsequent confirmation using whole-genome sequencing [51]. The prior for the specificity of PCR was based on values reported by Loy et al. [45] and modeled with a beta (31.4, 10.2) distribution for *M. haemolytica*, a beta (24.8, 7.8) distribution for *P. multocida*, and a beta (100, 5.6) distribution for *H. somni* [45].

The BLCM for macrolide resistance used uninformative priors modeled with beta [1] distributions for all parameters, due to a lack of comparable prior information for RPA, BC-AST, and qPCR. Convergence was assessed for all models, and deemed satisfactory if the evaluated model diagnostics, including potential scale reduction factor (PSRF < 1.05), effective sample size (>1000), and Monte Carlo standard errors as a percent of standard deviation (<5%), as well as visual inspection of trace and autocorrelation plots were satisfactory. Model parameters were estimated following 100,000 iterations of each of three Monte Carlo Markov chains, after a burn-in phase of 5000 iterations per chain. Estimates for test sensitivity and specificity were reported as the median of the posterior distribution along with 95% credible intervals (95% CrI). Of note for the BLCM estimating the performance of *M. haemolytica* detection, the RPA assay reported *M. haemolytica* serotypes 1 and 6 specifically, while bacterial culture and qPCR reported all serotypes.

Further agreement beyond that expected by chance between RPA, BC-AST, and qPCR for the detection of macrolide resistance or BRD-associated bacterial pathogens (RPA *M. haemolytica* assay serotypes 1, 6) in the DNPS was assessed using kappa (Stata/IC 15.1, StataCorp LLC, College Station, TX, USA). Kappa (κ) was interpreted as described by Dohoo, Martin, and Stryhn (κ ≤ 0.00—poor, 0.01–0.20—slight, 0.21–0.40—fair, 0.41–0.60—moderate, 0.61–0.80—substantial, 0.81–1.00—near perfect) [52] (pp. 97–99). Finally, odds ratios (Stata/IC 15.1) were calculated using two-by-two tables to describe the odds of detecting one or both macrolide ARG targets, given the presence of ICEs, in samples that were also positive or negative for *M. haemolytica*, *P. multocida*, and/or *H. somni*.

## 3. Results

### 3.1. Validation and Limit of Detection of RPA Assays for Macrolide Resistance Targets

The LOD for the singleplex RPA assay was 220 gc/reaction (95% CI (166, 371)) for the *msrE-mphE* assay, and 122 gc/reaction (95% CI (83, 412)) for the *erm42* assay. Once validated, the assays were combined at a 45 (*msrE-mphE*)-to-55 (*erm42*) primer and probe ratio, and validation of the multiplex macrolide resistance assay was completed using diluted genomic DNA from *M. haemolytica* MH44. The LODs of the multiplex assay were 168 gc/reaction (95% CI (130, 322)) for *msrE-mphE* and 185 gc/reaction (95% CI (144, 337)) for *erm42*.

### 3.2. Detection of Antimicrobial Resistance Determinants and Bacterial BRD Pathogens in DNPS

#### 3.2.1. Antimicrobial Resistance Gene Detection

The RPA assay detected *msrE*-*mphE* in 65% of samples, *erm42* in 1% of samples, and both gene targets in 4% of samples (Supplementary Table S3). From DNPS where at least one *M. haemolytica*, *P. multocida*, or *H. somni* isolate was resistant to macrolides (MacRes+, n = 101), *msrE* and/or *mphE* were detected in 95% of samples using RPA and 84% using PCR. Additionally, 41% of samples for RPA and 4–6% of samples for PCR were positive for *msrE* and/or *mphE* where target *Pasteurellaceae* bacteria not resistant to macrolides were identified by BC-AST (Table 5). Similarly, RPA identified *msrE*-*mphE* in 47% of samples where no target *Pasteurellaceae* bacteria were cultured. However, neither *msrE* nor *mphE* were identified by PCR in any of these samples (Table 5).

When parsed for the detection of *msrE* and/or *mphE* by RPA or PCR alone (Table 6), both *msrE* and *mphE* were typically detected or not detected as a pair by both tests. However, 3% of samples contained *mphE* without *msrE* as per PCR, where the same samples were positive for the *msrE-mphE* operon using RPA (Table 6). Detection of *erm42* using RPA was uncommon (4.5%) and no samples were positive for *erm42* using PCR (Table 5).

Based on the alignment of several partial bacterial sequences (Table 1 and Supplementary Materials—Figure S1, Geneious Prime v2020.0.4), the RPA and PCR primer/probe annealing sites for *msrE* and *mphE* differed. RPA primers targeted a highly conserved region of the *msrE-mphE* operon, and the previously validated PCR primers [46] annealed to a conserved region of *msrE* upstream. The PCR primer/probe annealing sites for *mphE* were downstream from the RPA reverse primer, and single nucleotide polymorphisms were observed in this region for one *M. haemolytica* and one *H. somni* isolate (Figure S1).

#### 3.2.2. BRD Pathogen Detection

Detection of BRD-associated bacteria varied across testing methods; RPA identified at least one bacterial pathogen of interest in 66% of samples. *M. haemolytica* of any serotype was identified in 73% of samples by culture and 74% of samples by qPCR, while RPA specifically detected serotypes 1 and 6 in 33% of samples (Table 7).

#### 3.2.3. ICE Detection

Samples positive for one or more of *M. haemolytica*, *P. multocida*, and/or *H. somni* using RPA (66%) were further tested for variants ICE*tnpA* and ICE*ebrB* (Table 8). Of these 132 samples, 38% possessed one or both variants. ICE*tnpA* was detected most frequently, in 70% (35/50) of ICE-positive samples, and 6% (3/50) of samples were found to carry both ICE variants.

Of the 132 samples positive for BRD organisms and tested for ICE, 94 were positive for *msrE*-*mphE* and/or *erm42* by RPA. Thirty-nine of these 94 ARG positive samples (41%) also harbored at least one ICE variant; *msrE*-*mphE* and/or *erm42* were detected by RPA in 78% (39/50) of samples with at least one detected ICE variant. Twenty-seven of the 50 ICE positive samples (54%) also had *M. haemolytica*, *P. multocida*, and/or *H. somni* isolates classified as phenotypically resistant to macrolides.

There was no significant association between the detection of either ICE variant by RPA in samples and the likelihood of identifying phenotypic macrolide resistance in the same samples (OR 1.21 (95% CI (0.63, 2.36)), *p* = 0.57) (n = 199). However, there was a significant association between detection of at least one ICE variant and detection of *msrE*-*mphE* and/or *erm42* by RPA (OR 7.44 (95% CI (3.37, 16.4)), *p* = 0.001) when only samples positive for *M. haemolytica*, *P. multocida*, or *H. somni* were considered (n = 199).

All samples ineligible for RPA testing for ICE and ARGs, as they were RPA negative for *M. haemolytica*, *P. multocida*, and *H. somni*, were considered negative in this serial testing strategy. The association between the detection of ICE and ARGs of interest was no longer significant if *msrE*-*mphE* and/or *erm42* detected by RPA were considered in all samples, regardless of the presence or absence of BRD pathogens as detected by RPA (OR 1.90 (95% CI (0.89, 4.10)), *p* = 0.10) (n = 199).

### 3.3. Diagnostic Performance Comparison

The clinical sensitivity of the real-time, multiplex RPA assay for *msrE*-*mphE* and *erm42* was estimated to be 95% and specificity was estimated to be 58% for all samples tested (Table 9). Sensitivity of culture was very similar to RPA and while the median sensitivity of PCR was slightly lower than RPA, the difference was not significant. However, the clinical specificity of both culture and qPCR were significantly higher (>95%) than that of RPA.

The same diagnostic testing strategy used for detection of ICEs was then applied to the detection of macrolide resistance genes by both RPA and qPCR in these samples, to investigate the effect on RPA specificity. Briefly, only samples positive for at least one BRD pathogen by RPA were tested for ARG by RPA, and similarly, only samples positive for at least one BRD pathogen using qPCR were tested for ARGs by qPCR. Samples that were not tested were considered negative for ARGs potentially associated with BRD pathogens. While this strategy improved the median specificity of RPA relative to the other tests, the increase was not significant (Table 9). Under this testing strategy, the sensitivity of RPA was significantly lower and was also significantly lower than BC-AST and qPCR (Table 9).

**Figure 2:**
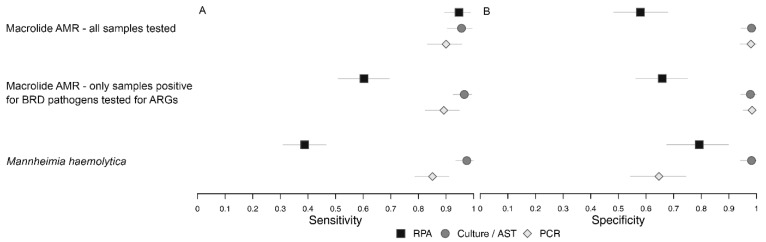
Plot of Bayesian latent class model estimates of sensitivity (**A**) and specificity (**B**) for models comparing detection of msrE-mphE +/− erm42 by recombinase polymerase amplification (RPA), macrolide resistance classified by antimicrobial susceptibility testing (AST), and detection of *msrE* OR *mphE* +/− *erm42* by polymerase chain reaction (PCR) when all samples were tested and when only samples positive for bovine respiratory disease bacteria were tested, as well as the model comparing detection of *M. haemolytica* by RPA (serotypes 1 and 6 only), culture (all serotypes) and PCR (all serotypes).

The differences in detection of macrolide resistance when only samples that were positive on RPA for at least one of the BRD organisms were tested (Table 9) as compared to when all samples were tested was summarized in Figure 2.

The test performance metrics from *M. haemolytica* were also included for context (Table 10), as most of the phenotypic macrolide resistance was described in this species (Table 3).

The qPCR test allowed for detection of either or both of *msrE* and *mphE* (“OR”). When the interpretation of the assay was changed so that both *msrE* and *mphE* (“AND”) had to be detected for the sample to be considered positive, the sensitivity of qPCR dropped slightly, and the RPA estimates were not impacted (Table 9).

Finally, the impact of how intermediate MICs were considered in the analysis was evaluated (Table 9). There was no observed difference in any of the estimates, except for a small but non-significant decrease in the specificity of culture and AST when intermediate MICs were considered together with resistant as “nonsusceptible”.

The results of Sanger sequencing were compared to those of bacterial culture, RPA, and PCR for an associated set of potential RPA false positives (n = 23; RPA-positive, and BC/AST- and qPCR-negative), to assess the lower RPA specificity for macrolide resistance genes (Table 4). Sanger sequencing for *msrE-mphE* did not confirm any false positives or false negatives for RPA. In contrast, the sequence quality of the eight samples tested for *erm42* was poor, and four false positives and 1 false negative were identified in these samples (Table 4 and Table S2).

The potential false positive samples subject to Sanger sequencing were also tested by long-read metagenomic sequencing, where *msrE* and/or *mphE* were identified in four samples. Of these, *mphE* was associated with a target BRD bacteria, *P. multocida*, in only one sample. In the remaining three samples, *msrE* and/or *mphE* genes were identified within reads of other bacterial species: *Moraxella bovoculi* (sample 2), *Citrobacter freundii* (sample 3), *Acinetobacter towneri* (sample 3), and *E. coli* (sample 3 and 4).

### 3.4. BRD Pathogen Sensitivity and Specificity Analysis

A similar Bayesian model was employed to estimate the clinical sensitivity and specificity of RPA, bacterial culture, and qPCR for the identification of the BRD pathogens of interest. RPA was insensitive to the choice of informative priors. Bayesian analysis of *M. haemolytica*, *P. multocida*, and *H. somni* across all three test types demonstrated highly variable sensitivities and specificities (Table 10), even though culture was highly specific for all three targets. For *M. haemolytica*, bacterial culture had the highest sensitivity. However, for both *P. multocida* and *H. somni* the sensitivity of qPCR was higher than for either culture or RPA. A two-test model for culture and qPCR for *M. haemolytica* generated almost identical specificity for both tests as compared to the three-test model that included RPA which only considered serotypes 1 and 6.

### 3.5. Agreement Among Assays

The agreement of RPA and AST or qPCR for the detection of macrolide resistance was moderate between RPA and both reference tests, and near perfect between AST and PCR (Table 11). The agreement between assays in the present study for the detection of organisms was fair between AST and qPCR and slight or poor for all other comparisons involving RPA (Table 11). The agreement for comparisons of qPCR and culture to RPA for identification of organisms tended to be lower for *P. multocida* and *H. somni*, where the assays evaluated the same target than for *M. haemolytica* where RPA considered only serotypes 1 and 6.

## 4. Discussion

Bovine respiratory disease remains the foremost reason for injectable antimicrobial use in Canadian feedlot cattle [53]. Macrolides are important for the management of both human and animal disease, and laboratory testing has been recommended to better inform antimicrobial use for BRD [8,54]. However, current diagnostic tools used to detect BRD bacterial pathogens and their antimicrobial resistance determinants are time-consuming and require specialized equipment. This two-part study developed and validated a novel, multiplex RPA assay targeting three clinically relevant macrolide resistance genes frequently associated with BRD bacterial pathogens. Development was supported by a curated isolate collection and DNA samples from DNPS collected from fall-placed calves that had been cultured and tested for antimicrobial susceptibility [41].

The assay enabled simultaneous and real-time detection of ARGs associated with macrolide resistance. The genetic linkage between *msrE* and *mphE* as an operon [2,14,16], supported by the sequencing data from isolates used in development, informed the design of the multiplex assay for three macrolide ARG targets (*msrE*, *mphE*, *erm42*). A combined assay for *msrE*-*mphE* was necessary for simultaneous analysis of all three genes, as the T16-ISO RPA machine was limited to two fluorescence channels. Nearly all samples (95%) where *M. haemolytica*, *P. multocida*, or *H. somni* isolates were phenotypically resistant to one of the macrolides were also positive for one or both RPA-targeted macrolide ARGs. In addition, the RPA targets for macrolide ARGs were detected in ~40% of samples where either the detected isolates were not phenotypically resistant to macrolides tested or no BRD-associated bacteria were detected.

Bayesian latent class modelling (BLCM) was used to estimate the diagnostic performance in the absence of a gold standard. RPA, AST, and qPCR all demonstrated good clinical sensitivity for macrolide ARGs and phenotypic macrolide resistance. However, RPA had lower specificity than AST and qPCR. In addition, both clinical sensitivity and specificity varied across all tests for identification of *M. haemolytica* (RPA: serotypes 1 and 6; culture/qPCR: all serotypes), *P. multocida*, and *H. somni*. The exceptions were the estimates for culture specificity, which were high (>97%) for all three bacterial targets.

BLCM validity relied on three assumptions: conditional independence of tests, differing prevalence of targets across populations, and consistent test performance across populations [55,56]. Culture and AST were considered independent from RPA and qPCR, which shared DNA extraction and some amplification methods. In the present study, samples collected at different times were expected to have different frequencies of both macrolide resistance and organisms of interest. Recent studies using the same calf data described varying prevalence of phenotypic macrolide resistance and *Pasteurellaceae* bacteria in cohorts of calves over time [40]. Capik et al. also found that isolation of BRD-related *Pasteurellaceae* from nasopharyngeal swabs varied over time in some calves treated for BRD [57]. Similarly, Holman et al. (2017) reported a significant shift over time in the recovery of *P. multocida* and *M. bovis* [58].

Although low RPA specificity suggested false positives, Sanger sequencing confirmed *msrE-mphE* in many suspect samples. Long-read metagenomic sequencing identified these genes in non-BRD bacteria, including *E. coli*, *Moraxella bovoculi*, *Citrobacter freundii*, and *Acinetobacter towneri* in three out of four suspect false-positive samples, as well as in *P. multocida* in one suspect RPA false-positive sample. This is consistent with conserved distribution of these ARGs across species such as *Acinetobacter* spp., *Staphylococcus aureus*, *Klebsiella pneumoniae*, *Escherichia coli*, *Moraxella osloensis*, and *Citrobacter freundii*, among others. These non-BRD-related species are common to humans, cattle, other animals, and the environment. Thus, RPA likely detected ARGs missed by BC-AST in part due to limited species testing.

Despite frequent use as a gold standard test, bacterial culture is imperfect and has limited sensitivity [59]. Further, phenotypic resistance cannot be confirmed without organism recovery. However, in the present study, bacterial recovery rates from DNPS from healthy, fall-placed calves were good, particularly for *M. haemolytica*, where most phenotypic macrolide resistance was identified. Overall *M. haemolytica* recovery rates ranged from 33% at 1DOF to 75% at 36 DOF [40], and compared well or were improved relative to similar reports [38,43,60], likely due to optimized sample handling. Pooled nasal swabs and prompt culture likely also improved recovery, as supported by studies emphasizing storage below 8 °C and culture within 24 h [61,62].

The culture recovery rates were reflected by very high estimated sensitivities from the BLCMs in the present analysis for both *M. haemolytica* and macrolide phenotypic resistance. Estimates of clinical sensitivity were lower for culture of both *P. multocida* and *H. somni*. Compared to *M. haemolytica* and *P. multocida*, *H. somni* has previously been reported to be challenging to culture [63]. In the present study, failure to culture target organisms and subsequently detect macrolide resistance could have affected RPA specificity estimates. Another potential factor could be that AST was completed on a single, purified colony per species per sample, a standard practice in diagnostic testing and field studies [38,41,43]. While necessary to manage logistics and cost, this approach also reflects how culture and AST are typically used to guide clinical decisions. However, testing only one colony presumes that all colonies of the same species on a plate share the same resistance profile [38,43]. This assumption could cause less prevalent, resistant subpopulations to be overlooked, particularly since the number of colonies needed to fully capture AMR diversity in a DNPS sample is not well defined [57,64,65]. In this study, although AST could have missed the detection of rare, resistant isolates of target bacteria [66,67], the high estimated sensitivity of BC-AST for detecting macrolide resistance suggested that the impact of this testing strategy was limited.

Despite the potential challenges associated with the sensitivity of culture and AST, the estimated low specificity of RPA for detecting macrolide ARGs was derived from a three-test Bayesian model that also included qPCR data. As a result, any potential limitations to the sensitivity of BC-AST would have been expected to impact the estimated specificity of qPCR and RPA in a similar manner. However, the qPCR specificity estimated from the same three-test BLCM was almost identical to that of culture, and significantly higher than the specificity of RPA. Both RPA and PCR interrogate DNA extracted from an entire sample. Therefore, the ARGs detected could have also been present in BRD-associated bacteria beyond the cultured species, such as *Bibersteinia trehalosi*, or in other microbiota commonly present in the bovine respiratory tract and the environment, such as *Moraxella* spp. or *Acinetobacter* spp. [68,69,70]. Interestingly, in the present study, the *msrE-mphE* operon was identified more frequently by RPA than *msrE* and/or *mphE* by qPCR in samples where no target *Pasteurellaceae* organisms were cultured (40% RPA versus 0% qPCR) and where macrolide-susceptible *Pasteurellaceae* were detected (41% RPA versus 4–6% qPCR).

Differences in RPA and qPCR assay performance might also reflect variations in primer design and assay thresholds. For example, the RPA primers targeted a highly conserved region of the *msrE-mphE* operon across *Pasteurellaceae* and other bacteria, whereas the validated PCR primers [46], specifically for *mphE*, annealed to a region more prone to single nucleotide polymorphisms in certain, rare gene variants of *M. haemolytica* and *H. somni*. This could be reflected in the median sensitivity of the qPCR assay, which was determined using the same DNA, but was estimated to be slightly lower than the sensitivity of RPA and AST.

These findings highlight a key diagnostic challenge: neither RPA nor qPCR can directly link detected ARGs to specific bacterial species of interest when applied to whole-sample DNA. To explore whether restricting interpretation of results could improve clinical relevance, macrolide ARG results were considered only in samples where the corresponding assay (RPA or qPCR) also detected *M. haemolytica*, *P. multocida*, or *H. somni*. This adjustment modestly increased the specificity of RPA but at the cost of a marked reduction in sensitivity. While these results supported the possibility that RPA detected macrolide ARGs in non-BRD-related bacteria, they also underscore the need for diagnostic tools that can resolve whether ARGs are associated with clinically relevant pathogens versus non-target microbiota.

Recognizing that some uncertainty remains in explaining why RPA detected *msrE-mphE* in samples missed by both BC-AST and qPCR, the Sanger sequencing data supporting these RPA-positive results was compelling. A possible contributing factor not evaluated in the present study was the physical configuration of the RPA reaction strip tube, which could allow microdroplet dispersal and cross-contamination between tubes in the T16-ISO instrument [71]. Although the tube containing the NTC was closed following the addition of the reaction mastermix, the other tubes typically remain open unless lids are cut and individually closed, creating a separate potential for contamination. While no identified publications have directly addressed this issue, Crannell et al. reported a similar, isolated issue during the development of an RPA-lateral flow assay for detection of *Cryptosporidium* spp. [72].

Contrary to the detection of *msrE-mphE*, the infrequent detection of *erm42* by RPA was less reliable. Sanger sequencing confirmed only a subset of results, with four of eight RPA-positive samples identified as false positive and one false negative. The low prevalence of *erm42* within the samples from this investigation made it difficult to evaluate assay performance, but was not surprising, as it was consistent with previous metagenomic and whole-genome sequencing studies of calf DNPS that identified abundant *msrE* and *mphE*, but no *erm42* [34,47]. Some other studies using PCR and whole-genome sequencing have identified a higher frequency of *erm42* as well as other ARGs harbored by *Pasteurellaceae* in both DNPS collected at feedlot arrival and pneumonic lung samples from BRD mortalities [2,13,14].

The interpretation of ARGs detected in this study was supported by parallel detection of ICEs by RPA. ICEs are known to harbor and transmit ARGs horizontally between members of the BRD bacterial complex, as well as bacteria unrelated to BRD [36]. In the present study, detection of at least one ICE variant was associated with the detection of *msrE-mphE*, but only within samples that were RPA-positive for one of the BRD bacteria. Previous investigations have reported macrolide resistance genes, along with other ARGs within ICE variants in BRD-associated bacteria [14,20,23,36,70,73,74,75]. However, DNA-target-based test methods like RPA and qPCR cannot link ICEs or ARGs to specific bacteria within a sample, and testing is limited to known ARG targets. In this study, five samples contained at least one of the key BRD-associated bacterial targets that were phenotypically resistant to macrolides, but *msrE-mphE* or *erm42* were not detected by RPA. In this case, it is possible that resistance might instead have been facilitated by genes that were recently discovered or are yet to be identified. For example, Dhindwal et al. identified *estT*, a previously undescribed macrolide esterase that hydrolyzes and inactivates macrolide antimicrobials commonly used in veterinary medicine (e.g., tylosin, tilmicosin, tildipirosin) [76,77,78]. As more ARGs are detected, additional RPA targets will be needed to maximize testing efficiency.

During assay design, the study could have also been limited by the need to alter the fluorescence threshold for detection of *msrE-mphE* and *erm42.* While previous studies by Conrad et al. used a fluorescence threshold of ≥100–200 mV for ≥60 s for detection of BRD-related bacterial targets and ICEs using RPA, the real-time assay for macrolide ARG detection required a more stringent fluorescence threshold (≥400 mV for ≥60 s) to resolve low-level, false-positive signals [27,29]. While raising the fluorescence threshold to mitigate false positives could have impacted the proportion of false-negative samples, the high sensitivity of the RPA assay (95%) suggests that this would have a negligible impact.

The low clinical sensitivity of RPA for *M. haemolytica*, *P. multocida*, and *H. somni* detection resulted in a high risk of false-negative samples. Samples that tested negative for all BRD organisms were not tested for ICEs and would not be tested for ARGs either if the serial testing strategy was applied, resulting in a missed opportunity for downstream information on AMR determinants. For detection of *P. multocida* (29%) and *H. somni* (48%) a previous study by Conrad et al. found the sensitivity of RPA to be similarly low relative to culture [29]. This finding of low sensitivity for detection of BRD bacteria likely explained the substantial drop in sensitivity for *msrE-mphE*/*erm42* detection when a serial clinical testing strategy was applied to the RPA results. It also further limits the future use of serial testing to target clinical decisions on ARG and ICE detection in samples positive for BRD bacteria.

However, for *M. haemolytica*, the interpretation of the BLCM was limited as the RPA assay targeted serotypes 1 and 6, while AST and qPCR were not specific to any serotype. *M. haemolytica* serotypes 1 and 6 have been identified most frequently in cattle with BRD [79]. Serotype 2 is more common in healthy cattle [38,79]. This target mismatch would not be expected to affect the estimated specificity of RPA relative to culture and qPCR. It could, however, explain in part the clinical sensitivity of RPA, which was estimated to be <40%; samples with target DNA from serotype 2 *M. haemolytica* would not be detected. This focus on targeting serotypes most often associated with BRD was intentional, but would fail to detect all *M. haemolytica*, thus impacting RPA assay sensitivity.

## 5. Conclusions

Despite certain limitations, RPA offers advantages of speed for individual assays, relative ease of use, and minimal equipment requirements, making it suitable for use in a dedicated workspace with DNA extraction capacity. The high clinical sensitivity of the *msrE-mphE* RPA assay supports its use as a rapid screening tool to rule out macrolide resistance in nasal swabs from high-risk cattle. The interpretation of a positive test result, however, might be limited by the broad range of bacteria not associated with BRD where these genes have been detected. The results of the Sanger sequencing support the detection of the targeted *msrE-mphE* by RPA, but not always in the targeted organisms.

Large-scale application would be constrained by current equipment configurations as, like all targeted assays, RPA cannot capture resistance mediated by ribosomal mutations, or novel or untargeted ARGs [34,73,76]. As more genes and mechanisms of resistance are identified, expansion of assay panels to include emerging determinants, such as *estT*, will be essential. Additional studies applied to greater numbers of samples from different geographic areas will provide additional context around the generalizability of these tests and the estimates of their performance. Further work is also needed to evaluate the economics and practicality of multiplexing several RPA assays per sample in commercial feedlot settings.

## Figures and Tables

**Table 1:** List of bacterial strains and whole-genome sequence data used in this study to develop and validate a recombinase polymerase amplification assay for the detection of macrolide resistance genes.

Species Name	Strain	Accession Number	Macrolide Resistance Genotype	Reference
*Histophilus somni*	HS J230	--	*msrE*, *mphE*	McAllister lab, unpublished
*Mannheimia haemolytica*	MH007	NZ_JBDJJR000000000	*msrE*, *mphE*	[34]
*Mannheimia haemolytica*	MH017	NZ_JBDJJS000000000	*msrE*, *mphE*	[34]
*Mannheimia haemolytica*	MH026	NZ_JBDJJT000000000	*msrE*, *mphE*	[34]
*Mannheimia haemolytica*	L044A (MH44)	LFXY00000000	*msrE*, *mphE*, *erm42*	[35]
*Mannheimia haemolytica*	T2	LFXW00000000	Negative control	[35]
*Mannheimia haemolytica*	MH 1609	NZ_CP017520	*msrE*, *mphE*	Published internally (US Meat Animal Research Center, USDA/ARS)
*Mannheimia haemolytica*	MH A145	--	*msrE*, *mphE*	McAllister lab, unpublished
*Mannheimia haemolytica*	MH A222	--	*msrE*, *mphE*	McAllister lab, unpublished
*Mannheimia haemolytica*	MH G146 *	--	*msrE*, *mphE*	McAllister lab, unpublished
*Mannheimia haemolytica*	MH USDA-ARS-USMARC-186	NZ_CP023047	*msrE*, *mphE*	Published internally (Genetics, Breeding, and Animal Health Research Unit, USDA-ARS-USMARC)
*Mannheimia haemolytica*	MH USDA-ARS-USMARC-193	NZ_CP023043	*msrE*, *mphE*	Published internally (Genetics, Breeding, and Animal Health Research Unit, USDA-ARS-USMARC)
*Mannheimia haemolytica*	MH 1450	NZ_CP017541	*msrE*, *mphE*, *erm42*	Published internally (US Meat Animal Research Center, USDA/ARS)
*Mannheimia haemolytica*	MH 1576	NZ_CP017524	*msrE*, *mphE*, *erm42*	Published internally (US Meat Animal Research Center, USDA/ARS)
*Pasteurella multocida*	RV_PM08	--	*erm42*	Waldner lab, unpublished
*Pasteurella multocida*	RV_PM39	--	*erm42*	Waldner lab, unpublished
*Pasteurella multocida*	PM 3361	NZ_CP026861	*msrE*, *mphE*	[36]
*Pasteurella multocida*	PM C231	--	*msrE*, *mphE*	McAllister lab, unpublished
*Pasteurella multocida*	PM 36950	NC_016808	*msrE*, *mphE*, *erm42*	[37]
*Pasteurella multocida*	PM 60494	NZ_CP015558	*msrE*, *mphE*, *erm42*	Published internally (Genetics, Breeding, and Animal Health Research Unit, USDA-ARS-USMARC)
*Pasteurella multocida*	PM 60714	NZ_CP015573	*msrE*, *mphE*, *erm42*	Published internally (Genetics, Breeding, and Animal Health Research Unit, USDA-ARS-USMARC)
*Pasteurella multocida*	PM22	NZ_CP045724	*msrE*, *mphE*, *erm42*	McAllister lab, published internally (Agriculture and Agri-Food Canada, Lethbridge Research and Development Centre)

Strain-specific genomic information was accessed through the National Center for Biotechnology Information (NCBI) database (National Library of Medicine (NLM), Bethesda, MD, United States), and from unpublished whole-genome sequence data provided by the McAllister lab (Lethbridge Research Centre, Agriculture and Agri-Food Canada, Lethbridge, AB, Canada). * A C-to-A single nucleotide polymorphism was noted for *M. haemolytica* strain MH G146 at the 49th nucleotide position from the 5′ end of *mphE*.

**Table 2:** Summary of primer and probe sets used to identify bacterial pathogens, integrative and conjugative elements, and macrolide resistance genes, in DNA from deep nasopharyngeal swabs collected from feedlot calves.

Target	Gene	Primer Sequences	Amplicon Size (bp)	Fluorescence Probe Sequence ^1^	Assay Type	Reference
**Bacterial BRD pathogens**						
*Histophilus somni*	Hs_0116	Fwd:_CGTTTAATCCCATTGCGATCATTCCCCATT Rev:_ATACTATTGCATTCGGCGATTTTTCCGCTT	342	TATTCAAGTAGATGCAGATGGGCAGCATAAFHQAATTGATGTCAAGAA	Hs/Pm multiplex	[27]
*Mannheimia haemolytica* serotypes (1, 6)	*nmaA*	Fwd:_TCAAAATGGCTCCCTTAGTTGAGGGCTTTA Rev:_AGTGGTTGCTGTATCGCCATGAACAAAAAT	254	TTCTGCTATTTTAGAAAAAATTCAACCTGTFHQTGCCGAATACAAAC	Multiplex *	[27]
*Pasteurella multocida*	*kmt1*	Fwd:_GAACCGATTGCCGCGAAATTGAGTTTTATG Rev:_CGAACTCGCCACTTTTTGTTTCATTTGGAC	417	ATTATTTTATGGCTCGTTGTGAGTGGGCTTGFHGGQAGTCTTTTATTT	Hs/Pm multiplex	[27]
**Antimicrobial resistance determinants**						
ICE*tnpA* ^2^	*tetH_tnpA*	Fwd:_CATCCACTAACTACGGCGCTGACATATCAA Rev:_TTGGTCCCCTTTTATTTGCCTTTATTTATA	318	ACACTGGTGCGGGAGATTGAGGCGGGGCGTFTHQAGCCCTGTTTCAACCC	ICE*tnpA*/ICE*ebrB* multiplex	[27]
ICE*ebrB* ^2^	*tetH_ebrB*	Fwd:_GAAAAGGTCGATTTTTGGGGAATTGCGAGC Rev: * Same as *tetH_tnpA*	351	ATTGGCTTGATTTTGGCAGGTGTGATAATGAFGHAQACGCTGTCCAAAATGG	ICE*tnpA*/ICE*ebrB* multiplex	[29]
Macrolide ARGs ^3^	*msrE-mphE* operon	Fwd:_CGAGATCAAAGACCACAAAATCATCAAGAC Rev:_TTCTTTCTTGATTTGTTCCCTCATGCCATCAC	284	AGCCAATCTAATCCGAACATTAATTATFGAHCQCTTTAAAGGAAATTA	MM/E ^4^ multiplex	This study
Macrolide ARG ^3^	*erm42*	Fwd:_GGTGCACCATCTTACAAGGAGTCTTATAAATC Rev:_GCATGCCTGTCTTCAAGGTTTATATCTGTAAAGTC	176	TTTATTATATAAACCATTTTTCAAAACFAAHAQATTGCATAGCTTT	MM/E ^4^ multiplex	This study

* Note: The multiplex, real-time RPA assay targeting *M. haemolytica* also detected *M. bovis*, but these results were outside the scope of this manuscript. ^1^ F = fluorophore, H = tetrahydrofuran, Q = quencher, ^2^ ICE = integrative and conjugative element, ^3^ ARG = antimicrobial resistance gene, ^4^ MM = *msrE-mphE*, E = *erm42*.

**Table 3:** Phenotypic resistance patterns of macrolide-resistant, bovine-respiratory-disease-associated bacterial pathogens isolated from deep nasopharyngeal swabs collected from fall-placed calves (*n* = 101).

Bacterial Species and Number of Cultured Isolates (%)	Resistance Pattern	Number of Isolates per Resistance Pattern (%)
*Mannheimia haemolytica*: n = 99 * (98.0)	Pansusceptible	0 (0.0)
	GAM-TUL	80 (80)
	GAM-TIL-TUL	8 (8.1)
	TIL	5 (5.1)
	GAM-TILD-TIL-TUL	1 (1.0)
	TILD-TIL	1 (1.0)
	GAM-TIL	1 (1.0)
	TIL-TUL	1 (1.0)
	AMP-GAM-TIL-TUL	1 (1.0)
	AMP-GAM-TUL	1 (1.0)
*Histophilus somni*: n = 20 (19.8)	Pansusceptible	17 (85)
	TILD	1 (5.0)
	TILD-TUL *	1 (5.0)
	TUL *	1 (5.0)
*Pasteurella multocida*: n = 25 (24.8)	Pansusceptible	23 (92)
	AMP	1 (4.0)
	SPECT-TET	1 (4.0)

Pansusceptible: The bacterial isolate was susceptible to all tested antimicrobials; AMP: ampicillin; GAM: gamithromycin; SPECT: spectinomycin; TET: tetracycline; TIL: tilmicosin; TILD: tildipirosin; TUL: tulathromycin. * *Mannheimia haemolytica* was isolated from 99 of the 101 MacRes+ samples, from which either *M. haemolytica*, and/or *H. somni* demonstrated phenotypic macrolide resistance. *M. haemolytica* was not recovered from the remaining two samples; instead, macrolide resistance was associated with *H. somni* isolates resistant to TILD-TUL and TUL, respectively.

**Table 4:** Sanger sequencing of purported false-positive recombinase polymerase amplification DNA products for the alignment of primer and probe sets associated with detection of macrolide resistance genes *msrE-mphE* and *erm42*, compared to the results of antimicrobial susceptibility testing and polymerase chain reaction testing (*n* = 31).

Macrolide Antimicrobial Resistance Gene Target	Test Result Combination by Antimicrobial Resistance Gene Target						Sanger Sequencing Results	RPA Result Compared to Sanger Sequencing			
	AST ^1^	RPA ^2^ (*msrE-mphE* *)	RPA (*erm42*)	PCR ^3^ (*msrE* *)	PCR (*mphE* *)	PCR ** (*erm42*)	Number of positive samples using Sanger sequencing **	RPA TP ^4^	RPA FP ^5^	RPA TN ^6^	RPA FN ^7^
*msrE*, *mphE* (n = 23)	-	+		-	-		23	23	0	0	0
*erm42* (n = 2)	+		+			-	0	0	2	0	0
*erm42* (n = 3)	+		-			-	1	0	0	2	1
*erm42* (n = 3)	-		+			-	1	1	2	0	0

* The RPA multiplex assay for macrolide resistance targeted *msrE* and *mphE* simultaneously as an operon, while the qPCR multiplex assay targeted each gene individually. ** For this comparison, Sanger sequencing was treated as the gold standard reference test for comparing the results of BC-AST, RPA, and qPCR testing, and was assumed to be 100% specific. ^1^ AST: antimicrobial susceptibility testing; ^2^ RPA: recombinase polymerase amplification; ^3^ PCR: polymerase chain reaction; ^4^ TP: true positive; ^5^ FP: false positive; ^6^ TN: true negative; ^7^ FN: false negative.

**Table 5:** Summary of the reported phenotypic macrolide resistance compared to macrolide resistance genes detected by recombinase polymerase amplification and real-time polymerase chain reaction in DNA extracted from deep nasopharyngeal swabs from fall-placed calves (*n* = 199).

		Samples Positive for (%):				
		*msrE-mphE* (RPA ^1^)	*erm42* (RPA)	*msrE* or *mphE* (PCR ^2^)	*msrE* and *mphE* (PCR)	*erm42*, *msrE*, or *mphE* (RPA or PCR)
**Culture result and macrolide** **resistance** **phenotype**	MH ^3^, PM ^4^, or HS ^5^ isolated Resistant (n = 101)	96 (95.0)	2 (2.0)	90 (89.1)	85 (84.2)	97 (96.0)
	MH, PM, or HS isolated Susceptible (n = 68)	28 (41.2)	3 (4.4)	4 (5.9)	3 (4.4)	28 (41.2)
	MH, PM, or HS Not isolated (n = 30)	12 (40.0)	4 (13.3)	0 (0.0)	0 (0.0)	14 (46.7)
**Macrolide ARG ^6^-positive samples**		136 (68.3)	9 (4.5)	94 (47.2)	88 (44.2)	139 (69.8)

^1^ RPA: recombinase polymerase amplification; ^2^ PCR: polymerase chain reaction; ^3^ MH: *M. haemolytica*; ^4^ PM: *P. multocida*; ^5^ HS: *H. somni*; ^6^ ARG: antimicrobial resistance gene. Extracted DNA from 199 deep nasopharyngeal swab samples selected based on antimicrobial susceptibility data were further tested by qPCR, targeting macrolide resistance genes *msrE*, *mphE*, and/or *erm42* individually, and RPA, which targeted *erm42* individually and *msrE-mphE* together as an operon. The latter genes (*msrE* and/or *mphE*) were detected in various combinations by either test method in 137 samples. Of the samples that tested negative for *msrE-mphE* by RPA (n = 63), bacteria phenotypically resistant to macrolides were detected in five samples, and one of these samples was also positive for *msrE* and *mphE* by PCR. *Erm42* was not detected in the 199 samples tested by PCR.

**Table 6:** Summary of macrolide resistance genes detected by recombinase polymerase amplification and real-time polymerase chain reaction in DNA extracted from deep nasopharyngeal swabs from fall-placed calves (*n* = 199).

		*msrE* (PCR ^2^)				
		Detected		Not Detected		
		*mphE *(PCR)		*mphE *(PCR)		
		Detected (%)	Not Detected (%)	Detected (%)	Not Detected (%)	
***msrE-mphE* (RPA ^1^)**	Detected	87 (43.7)	0 (0.0)	6 (3.0)	43 (21.6)	136 (68.3)
	Not detected	1 (0.5)	0 (0.0)	0 (0.0)	62 (31.2)	63 (31.7)
		88 (44.2)	0 (0.0)	6 (3.0)	105 (52.8)	199 (100)

^1^ RPA: recombinase polymerase amplification; ^2^ PCR: polymerase chain reaction. *mphE* was detected by PCR without *msrE* in six samples, while the same samples were positive for the *msrE-mphE* operon using RPA.

**Table 7:** Frequency of detection of bovine-respiratory-disease-associated bacterial pathogens by recombinase polymerase amplification, bacterial culture, and quantitative polymerase chain reaction (*n* = 199).

	Count of Positive Samples by Test Method		
Bacterial Species Name	RPA ^2^ (%)	Culture (%)	PCR ^3^ (%)
*Mannheimia haemolytica* ^1^	66 (33.2)	145 (72.9)	148 (74.4)
*Pasteurella multocida*	66 (33.2)	35 (17.6)	102 (51.3)
*Histophilus somni*	72 (36.2)	53 (26.6)	93 (46.7)

^1^ The RPA assay targeting *M. haemolytica* specifically detected serotypes 1 and 6, which are more commonly associated with disease, while bacterial culture and the completed qPCR assay isolated/targeted all MH serotypes. ^2^ RPA: recombinase polymerase amplification; ^3^ PCR: polymerase chain reaction.

**Table 8:** Prevalence of integrative and conjugative element (ICE) variants and bovine-respiratory-disease (BRD) bacteria detected by recombinase polymerase amplification in samples positive for at least one species of *Mannheimia haemolytica*, *Pasteurella multocida*, or *Histophilus somni* (*n* = 132 *).

	Count of ICE-Positive Samples (% of Samples with BRD Bacteria) *	Count of ICE-Positive Samples (% of ICE-Positive Samples), by Bacterial Species:		
ICE Variant Target		*M. haemolytica* ^1^	*P. multocida*	*H. somni*
ICE*tnpA* only	32 (24)	25 (78)	13 (41)	12 (38)
ICE*ebrB* only	15 (11)	5 (33)	12 (80)	7 (47)
ICE*tnpA* and ICE*ebrB*	3 (2.3)	1 (33)	2 (67)	1 (33)
Total ICE-positive samples	50 (38)	31 (62)	27 (54)	20 (40)

* RPA testing for ICEs was completed only on samples where any of *M. haemolytica*, *P. multocida*, or *H. somni* were detected using RPA (66.3%, 132/199). ICE*tnpA*: gene targets *tetH_tnpA*; ICE*ebrB*: gene targets *tetH_ebrB*. ^1^ The RPA assay for *M. haemolytica* specifically targeted serotypes 1 and 6.

**Table 9:** Clinical sensitivity and specificity of recombinase polymerase amplification, bacterial culture and antimicrobial susceptibility testing, and polymerase chain reaction for detection of macrolide antimicrobial resistance genes in deep nasopharyngeal swabs obtained from feedlot calves, based on Bayesian latent class models (*n* = 199).

Antimicrobial Resistance Gene and Target Test	Sensitivity * (95% CrI)	Specificity * (95% CrI)
**Macrolide resistance—all samples tested**		
RPA ^1^ (*msrE-mphE* +/− *erm42*)	94.7% (89.5%, 98.9%)	58.0% (48.3%, 67.9%)
Bacterial culture and AST ^2^ (Phenotypic macrolide resistance)	95.6% (90.5%, 99.4%)	98.2% (94.4%, 99.99%)
PCR ^3^ (*msrE* **OR** *mphE* +/− *erm42*)	90.0% (83.3%, 95.7%)	98.0% (94.1%, 99.99%)
**Macrolide resistance—only samples positive for BRD pathogens eligible for ARG testing**		
RPA ^1^ (*msrE-mphE* +/− *erm42*)	60.3% (50.9%, 69.5%)	65.9% (56.4%, 75.0%)
Bacterial culture and AST ^2^ (Phenotypic macrolide resistance)	96.6% (92.6%, 99.3%)	97.8% (94.3%, 99.99%)
PCR ^3^ (*msrE* **OR** *mphE* +/− *erm42*)	89.2% (82.5%, 94.8%)	98.4% (95.2%, 99.99%)
**Macrolide resistance—all samples tested**		
RPA ^1^ (*msrE-mphE* +/− *erm42*)	94.8% (90.0%, 98.7%)	57.4% (47.6%, 67.0%)
Bacterial culture and AST ^2^ (Phenotypic macrolide resistance)	96.0% (91.2%, 99.4%)	97.5% (93.4%, 99.99%)
PCR ^3^ (*msrE* **AND** *mphE* +/− *erm42*)	85.5% (78.1%, 92.1%)	98.6% (95.5%, 99.99%)
**Macrolide resistance—all samples tested-alternate classification of phenotypic susceptibility**		
RPA ^1^ (*msrE-mphE* +/− *erm42*)	95.2% (90.1%, 99.5%)	58.7% (48.7%, 68.2%)
Bacterial culture and AST ^2^ (Phenotypic macrolide non-susceptible) ^4^	95.9% (90.8%, 99.3%)	94.4% (88.5%, 98.7%)
PCR ^3^ (*msrE* **OR** *mphE* +/− *erm42*)	90.5% (84.0%, 96.5%)	98.9% (95.7%, 99.99%)

* The sensitivity and specificity of the RPA assays were estimated using a Bayesian latent class model comparing three tests and three populations. ^1^ recombinase polymerase amplification (RPA); ^2^ antimicrobial susceptibility testing (AST); ^3^ polymerase chain reaction (PCR). ^4^ Antimicrobial phenotypes classified as intermediate and resistant compared to susceptible.

**Table 10:** Clinical sensitivity and specificity of recombinase polymerase amplification (RPA), bacterial culture, and polymerase chain reaction (PCR) for the detection of bovine-respiratory-disease bacteria in deep nasopharyngeal swabs obtained from feedlot calves, based on Bayesian latent class analysis (*n* = 199).

Bacterial Species Name and Target Test	Sensitivity * (95% CrI)	Specificity * (95% CrI)
***Mannheimia haemolytica* ^1^**		
RPA (serotypes 1, 6)	38.8% (31.0%, 46.6%)	79.3% (67.5%, 89.9%)
Bacterial culture (all serotypes)	97.5% (93.6%, 99.99%)	98.2% (94.2%, 99.99%)
PCR (all serotypes)	85.1% (78.7%, 91.0%)	64.7% (54.4%, 74.5%)
***Pasteurella multocida***		
RPA	36.5% (28.8%, 44.7%)	68.4% (60.8%, 76.2%)
Bacterial culture	32.8% (21.0%, 46.1%)	97.5% (94.5%, 99.99%)
PCR	90.8% (77.2%, 99.99%)	87.0% (74.4%, 95.9%)
***Histophilus somni***		
RPA	44.7% (34.9%, 55.1%)	77.1% (62.7%, 95.2%)
Bacterial culture	41.6% (31.0%, 53.0%)	98.9% (96.2%, 99.99%)
PCR	68.6% (56.2%, 81.2%)	93.6% (87.0%, 98.2%)

* The sensitivity and specificity of the RPA assays were estimated using a Bayesian latent class model comparing three tests and three populations. ^1^ The RPA assay for *M. haemolytica* specifically targeted serotypes 1 and 6, which are more commonly associated with disease, while bacterial culture and qPCR were not serotype-specific.

**Table 11:** Inter-test agreement (kappa, κ) between recombinase polymerase amplification, bacterial culture, antimicrobial susceptibility testing, and/or quantitative polymerase chain reaction for detection of bovine-respiratory-disease-associated bacteria and macrolide antimicrobial resistance genes in deep nasopharyngeal swab samples obtained from feedlot calves (*n* = 199).

Antimicrobial Resistance Type or Bacterial Species Name	Test Combination	Two Tests (95% CI) (Cohen’s Kappa)
Macrolide resistance ^1,2,3,4^	RPA ^4^, AST ^2^	0.52 (0.41, 0.64)
Macrolide resistance ^1,2,3,4^	RPA ^4^, qPCR ^3^	0.55 (0.44, 0.65)
Macrolide resistance ^1,2,3,4^	qPCR ^3^, AST ^2^	0.85 (0.78, 0.92)
*Mannheimia haemolytica* *	RPA ^4^, BC ^1^	0.10 (0.01, 0.20)
*Mannheimia haemolytica* *	RPA ^4^, qPCR ^3^	0.08 (0.00, 0.18)
*Mannheimia haemolytica* *	BC ^1^, qPCR ^3^	0.39 (0.25, 0.54)
*Pasteurella multocida*	RPA ^4^, BC ^1^	0.00 (0.00, 0.08)
*Pasteurella multocida*	RPA ^4^, qPCR ^3^	0.00 (0.00, 0.13)
*Pasteurella multocida*	BC ^1^, qPCR ^3^	0.24 (0.14, 0.34)
*Histophilus somni*	RPA ^4^, BC ^1^	0.09 (0.00, 0.23)
*Histophilus somni*	RPA ^4^, qPCR ^3^	0.00 (0.00, 0.12)
*Histophilus somni*	BC ^1^, qPCR ^3^	0.23 (0.11, 0.36)

* The RPA assay for *M. haemolytica* specifically targeted serotypes 1 and 6, while traditional culture methods and the real-time PCR assay were not serotype-specific. ^1^ Bacterial culture (BC) comprised the isolation of specific bacterial species important to bovine respiratory disease, while ^2^ antimicrobial susceptibility testing (AST) determined if a BRD-associated isolate showed phenotypic resistance to macrolides tulathromycin, tilmicosin, tildipirosin, and/or gamithromycin. ^3^ Multiplex, quantitative, real-time polymerase chain reaction assays (qPCR) and ^4^ multiplex, real-time recombinase polymerase amplification (RPA) assays targeted *M. haemolytica*, *P. multocida*, and *H. somni*, or key macrolide resistance genes (*msrE* and *mphE* +/− *erm42* by qPCR and *msrE-mphE* and/or *erm42* by RPA).

## Data Availability

The original contributions presented in this study are included in the article/Supplementary Materials. Further inquiries can be directed to the corresponding author(s).

## Supplementary Materials

The following supporting information can be downloaded at: https://www.mdpi.com/article/10.3390/vetsci12111079/s1. Table S1: Phenotypic resistance patterns of bacterial bovine respiratory disease pathogens *Mannheimia haemolytica*, *Pasteurella multocida*, and *Histophilus somni* isolated from deep nasopharyngeal swabs, which were susceptible to macrolide antimicrobials (n = 68). Table S2: Results of Sanger sequencing of recombinase polymerase amplification DNA products for the alignment of associated primer and probe sets for *msrE-mphE* and *erm42*, compared to different combinations of results of antimicrobial susceptibility testing and polymerase chain reaction (n = 48). Table S3: Summary of macrolide resistance genes detected by recombinase polymerase amplification and real-time polymerase chain reaction in DNA extracted from deep nasopharyngeal swabs (n = 199). Table S4: Select list of bacterial species identified by metagenomic sequencing to contain macrolide resistance genes msrE and/or mphE (preliminary findings). Figure S1: Alignment of recombinase polymerase amplification and polymerase chain reaction primers and probes for detection of macrolide resistance genes *msrE* and/or *mphE*.
