# Supplementary material for: Novel Recombinase Polymerase Amplification Assay Is Sensitive for Detection of Macrolide Resistance Genes Relevant to Bovine Respiratory Disease Management in Feedlot Calves
Source: Vet Sci. 2025 Nov 12;12(11):1079. doi: 10.3390/vetsci12111079 (PMC12656793; doi:10.3390/vetsci12111079)
Supplement: Supplementary file 1 [file vetsci-12-01079-s001.zip › vetsci-3938108-supplementary.pdf]

Supplementary Materials

Novel recombinase polymerase amplification assay is sensitive for detection of macrolide resistance genes relevant to bovine respiratory disease management in feedlot calves

Tara Funk, Lianne McLeod, Cheyenne C. Conrad, Rahat Zaheer, Simon J. G. Otto, Cheryl L. Waldner*, and Tim A. McAllister

*Correspondence: cheryl.waldner@usask.ca

DNA copy number calculation

The DNA copy number calculation (i.e. the number of genome copies per microlitre) was determined using the following equation:

$$\textit{Number of genome copies} = \frac{\textit{Amount of DNA}\ (\text{ng}) * 6.022 \text{ x } 10^{23}\ \text{mol}^{-1}}{(\textit{Length}\ (\text{bp}) * 660\ \text{g/mol}) * 1 \text{ x } 10^{9}\ \text{ng/g}}$$

Where,
$6.022 \text{ x } 10^{23}$ = Avogadro's constant, expressed in mol^{-1}
Length (bp) = length of the dsDNA amplicon or genome in base pairs
660 g/mole = average mass of 1 bp of dsDNA
$1 \text{ x } 10^{9}$ ng/g = conversion factor for nanograms to grams

Table S1: Phenotypic resistance patterns of bacterial bovine respiratory disease pathogens Mannheimia haemolytica, Pasteurella multocida, and Histophilus somni isolated from deep nasopharyngeal swabs, which were susceptible to macrolide antimicrobials (n = 68).

**Bacterial species name and number of cultured isolates (%)**	**Resistance pattern**	**Number of isolates per resistance pattern (%)**
Mannheimia haemolytica: 33 (48.5)	Pansusceptible	32 (97.0%)
	AMP	1 (3.0%)
Pasteurella multocida: 52 (76.5)	Pansusceptible	43 (82.7%)
	SP-TET	9 (17.3%)
Histophilus somni: 6 (8.8)	Pansusceptible	6 (100)

Pansusceptible: The bacterial isolate was susceptible to all tested antimicrobial drugs; AMP: ampicillin; SP: spectinomycin; TET: tetracycline.

Table S2: Results of Sanger sequencing of recombinase polymerase amplification DNA products for the alignment of associated primer and probe sets for msrE-mphE and erm42, compared to different combinations of results of antimicrobial susceptibility testing and polymerase chain reaction (n = 48).

**Macrolide antimicrobial resistance gene target**	**Test result combination by antimicrobial resistance gene target**						**Sanger sequencing results**	**RPA result compared to Sanger sequencing**				**Additional comments regarding other test methods**
	BC-AST	RPA (MM*)	RPA (*erm42*)	PCR (*msrE*)	PCR (*mphE*)	PCR** (*erm42*)	Number of positive samples using Sanger sequencing	RPA TP	RPA FP	RPA TN	RPA FN	
msrE, mphE (n = 3)	+	+		+	+		3	3	0	0	0	
msrE, mphE (n = 3)	+	+		-	-		3	3	0	0	0	PCR *msrE, mphE* false negative (3)
msrE, mphE (n = 3)	+	+		-	+		3	3	0	0	0	PCR *msrE* false negative (3)
msrE, mphE (n = 1)	+	-		+	+		1	0	0	0	1	
msrE, mphE (n = 3)	-	+		+	+		3	3	0	0	0	BC-AST macrolide resistance false negative (3)
msrE, mphE (n = 1)	-	+		-	+		1	1	0	0	0	BC-AST macrolide resistance and PCR *msrE* false negative (1)
msrE, mphE (n = 3)	+	-		-	-		2	0	0	1	2	PCR *msrE, mphE* false negative (2)
msrE, mphE (n = 24)	-	+		-	-		24	24	0	0	0	BC-AST macrolide resistance and PCR *msrE, mphE* false negative (24)
erm42 (n = 2)	+		+			-	0	0	2	0	0	BC-AST macrolide resistance false positive (2)

**Macrolide antimicrobial resistance gene target**	**Test result combination by antimicrobial resistance gene target**						**Sanger sequencing results**	**RPA result compared to Sanger sequencing**				**Additional comments regarding other test methods**
	BC-AST	RPA (MM*)	RPA (*erm42*)	PCR (*msrE*)	PCR (*mphE*)	PCR** (*erm42*)	Number of positive samples using Sanger sequencing	RPA TP	RPA FP	RPA TN	RPA FN	
erm42 (n = 3)	+		-			-	1	0	0	2	1	PCR *erm42* false negative (1); BC-AST macrolide resistance false positive (2)
erm42 (n = 3)	-		+			-	1	1	2	0	0	BC-AST macrolide resistance and PCR *erm42* false negative (1)

Total samples tested for *msrE-mphE (*n = 41)
Total samples tested for *erm42 (*n = 8)
Total samples tested for macrolide ARGs (n = 48*)**

**The RPA multiplex assay for genotypic macrolide resistance targeted msrE and mphE (MM) simultaneously as an operon. **Of all the samples tested (n = 199), none were positive for erm42 by PCR. ***One sample was submitted twice for Sanger sequencing, as it tested positive for both macrolide resistance gene targets (msrE-mphE, erm42) by RPA, resulting in 48 total samples tested instead of 49. Abbreviations: AST = antimicrobial susceptibility testing; BC = bacterial culture; PCR = polymerase chain reaction; RPA = recombinase polymerase amplification testing; RPA FN = RPA false negative; RPA FP = RPA false positive; RPA TN = RPA true negative; RPA TP = RPA true positive.*

In total, a subset of 48 samples tested for macrolide ARGs were submitted to the National Research Council (NRC) of Canada (Saskatoon, SK, Canada) for Sanger sequencing (Supplementary material – Table S2). These samples were specifically selected for further sequencing because they showed different combinations of results from BC-AST, RPA, and qPCR testing, each bringing into question the validity of the RPA results (i.e. potential false positives).

Table S3: Summary of macrolide resistance genes detected by recombinase polymerase amplification and real-time polymerase chain reaction in DNA extracted from deep nasopharyngeal swabs (n = 199).

Test method	Macrolide ARG[1] detected	Count of positive samples (%)	Total count of macrolide-ARG[1]-positive samples (%)
RPA[2]	*msrE-mphE* only	129 (65)	138 (69)
	erm42 only	2 (1.0)	
	msrE-mphE and *erm42*	7 (3.5)	
PCR[3]	*msrE* only	0 (0.0)	94 (47)
	mphE only	6 (3.0)	
	msrE and *mphE* only	88 (44)	
	erm42 only	0 (0.0)	
	msrE, mphE, and *erm42*	0 (0.0)	

*[1]ARG: antimicrobial resistance gene; [2]RPA: recombinase polymerase amplification; [3]PCR: polymerase chain reaction. *The qPCR assay targeted macrolide resistance genes msrE, mphE, and erm42 individually, while RPA targeted msrE-mphE together as an operon.*

Table S4: Select list of bacterial species identified by metagenomic sequencing to contain macrolide resistance genes msrE and/or mphE (preliminary findings).

Resistance gene	Bacterial species name	Corresponding sample number
msrE	*Moraxella bovoculi*	2046A-1-076
	Escherichia coli	2048B-2-088
	Citrobacter freundii	2048B-2-088
	Acinetobacter towneri	2048B-2-088
mphE	*Pasteurella multocida**	2046B-1-018
	Escherichia coli	2048B-2-088
msrE and *mphE*	*Escherichia coli*	2047A-1-040
	Escherichia coli	2047A-1-040

**The only sample where metagenomic sequencing identified an mphE-positive Pasteurellaceae organism that had been missed by bacterial culture/antimicrobial susceptibility testing and qPCR.*

Figure S1: Alignment of recombinase polymerase amplification and polymerase chain reaction primers and probes for detection of macrolide resistance genes msrE and/or mphE.

A.

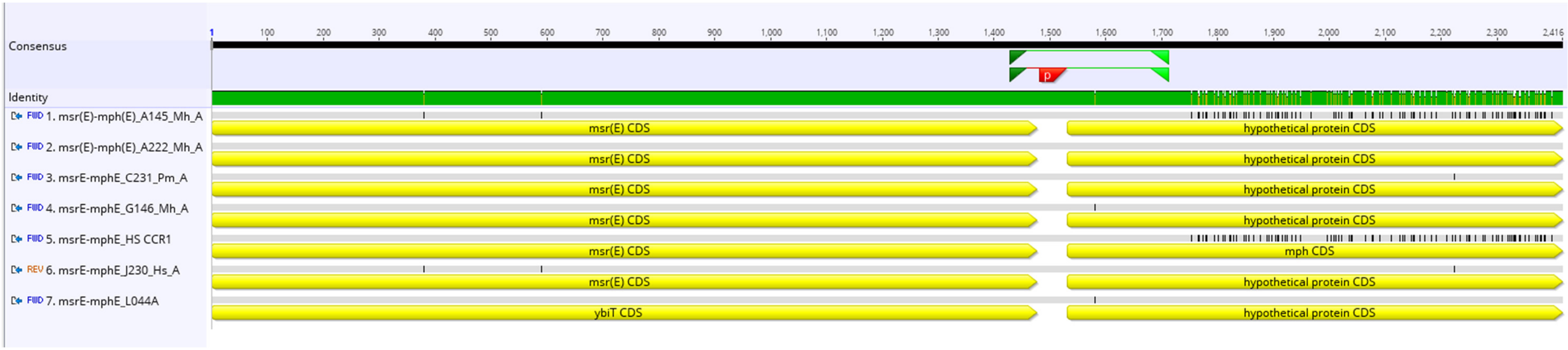

B.

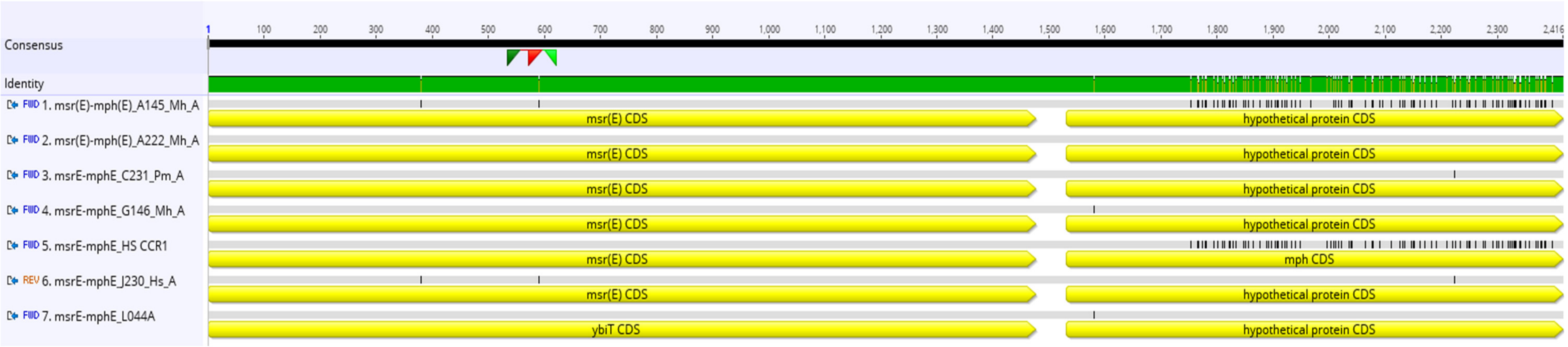

Figure S1 (continued): Alignment of recombinase polymerase amplification and polymerase chain reaction primers and probes for detection of macrolide resistance genes msrE and/or mphE.

C.

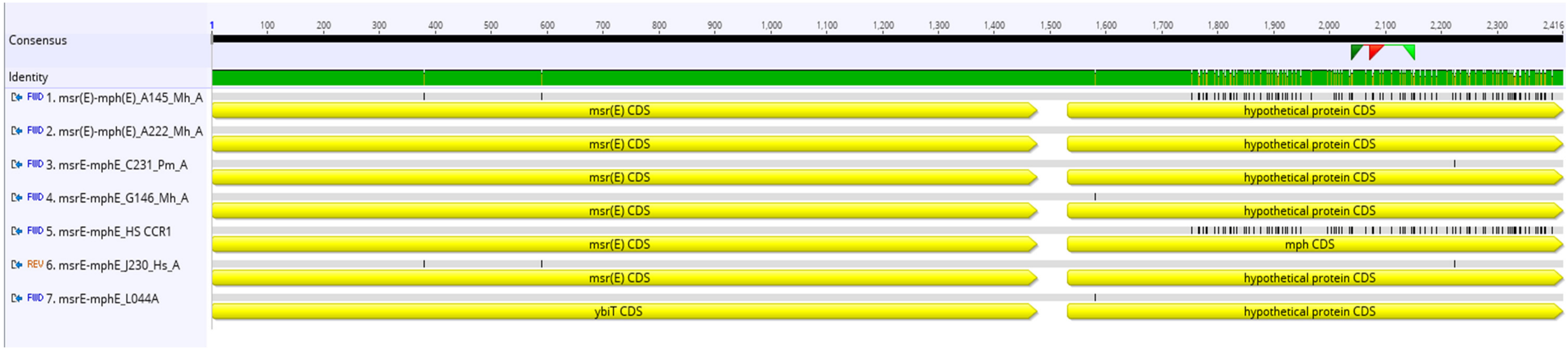

Alignment of partial genomic sequences, including primers and probes for detection of A) msrE-mphE by RPA, B) msrE by qPCR, and C) mphE by qPCR. In some isolates, the mphE gene was denoted by the generalized term of "hypothetical protein". In addition to the genomic sequences used for RPA primer and probe development, as referenced in Table 1 of the present study, the sequence for H. somni CCR1 (accession number: NZ_CP186878) contained numerous single nucleotide polymorphisms at the annealing site of the qPCR primers and probes targeting mphE, whereas the annealing sites for msrE (qPCR) and the msrE-mphE operon (RPA) appeared to be highly conserved. All other sequences are referenced in Table 1 of the present study.
